# Additional Fracture Detection by Computed Tomography After Plain Radiography in Adults with Isolated Foot and Ankle Trauma: A Retrospective Selected-Cohort Study

**DOI:** 10.3390/jcm15166214

**Published:** 2026-08-11

**Authors:** Adnan Arslan, Aytekin Dikici, Ferhat Danışman, Yunus Can Ünal, Ömer Faruk Yıldırım, Şehmuz Kaya

**Affiliations:** 1 Department of Orthopaedics and Traumatology, Van Training and Research Hospital, 65300 Van, Türkiye; fth30arslan@gmail.com (A.A.); aytekindkc@hotmail.com (A.D.); dr.frht@hotmail.com (F.D.);; 2 Department of Orthopaedics and Traumatology, Faculty of Medicine, Van Yuzuncu Yil University, 65080 Van, Türkiye; sehmuzkaya@doctor.com

**Keywords:** foot trauma, ankle trauma, computed tomography, plain radiography, occult fracture, diagnostic imaging, fracture detection

## Abstract

**Background/Objectives:** Plain radiography is the first-line imaging modality for acute foot and ankle trauma; however, computed tomography (CT) may provide additional information in selected patients with persistent clinical suspicion, equivocal radiographic findings, or a need for detailed fracture characterization. We hypothesized that the CT-confirmed fracture proportion would be higher among patients with suspicious radiographs than among those with normal radiographs and that CT would identify additional fractures in a clinically selected cohort. **Methods:** This retrospective single-center selected-cohort study included 1000 adult patients with isolated foot and/or ankle trauma who underwent both plain radiography and CT during the same clinical encounter. Plain radiographs were categorized as normal, suspicious for fracture, or definite fracture on the basis of original radiology reports and available imaging records. A CT-confirmed fracture was defined as the presence of an acute fracture on CT. Analyses focused primarily on CT-confirmed fracture proportions and conditional diagnostic yield within the selected CT cohort. Conditional apparent diagnostic performance was evaluated as a supplementary sensitivity analysis and was not intended to estimate population-level diagnostic accuracy. **Results:** Plain radiographs were categorized as normal in 628 patients, suspicious in 283, and definite fracture in 89. CT detected acute fractures in 386 patients (38.6%). The CT-confirmed fracture proportions were 14.8% in the normal radiograph group, 72.1% in the suspicious radiograph group, and 100.0% in the definite fracture group. The proportion of patients without a definite fracture on plain radiography but with an acute fracture on CT was 29.7%. Fracture-pattern classification was unavailable in 85 of 386 cases with CT-confirmed fractures (22.0%); therefore, fracture-pattern analyses were considered exploratory. **Conclusions:** In this selected CT cohort, CT identified additional acute fractures in a subset of adult isolated foot and ankle trauma cases that could not be definitively classified as fractures on plain radiography. Because CT was not systematically performed in all trauma patients, the findings represent selected-cohort CT yield rather than true population-level diagnostic accuracy.

## 1. Introduction

Foot and ankle trauma is among the most common musculoskeletal injury presentations in emergency and orthopedic practice. Early and accurate identification of clinically relevant fractures is important for treatment selection, preservation of articular congruity, avoidance of missed injuries, and prevention of delayed morbidity. Current American College of Radiology (ACR) Appropriateness Criteria define plain radiography as the basic first-line imaging modality for acute foot and ankle trauma, while advanced imaging is reserved for selected scenarios in which radiographic findings are inconclusive or more detailed anatomic characterization is needed [1,2].

Plain radiography is rapid, widely available, inexpensive, and usually sufficient for the initial assessment of foot and ankle trauma. Nevertheless, radiographic interpretation can be limited by overlapping osseous structures, complex regional anatomy, subtle cortical disruption, trabecular injury, minimal displacement, and equivocal findings near the articular surface. Radiographically occult and subtle fractures have been described across musculoskeletal trauma, and persistent focal pain or discordance between clinical examination and radiographic findings may justify additional imaging in selected patients [3,4,5].

The role of CT is particularly relevant in anatomic regions where fracture morphology can influence clinical management. Previous studies have shown that CT may improve characterization of Lisfranc injuries, talar fractures, calcaneal fractures, posterior malleolar fractures, and complex malleolar or syndesmotic injuries [6,7,8,9,10,11]. These studies support the clinical value of CT in selected situations, especially for defining intra-articular extension, comminution, fragment configuration, and preoperative planning. However, many available investigations focus on specific fracture types, relatively small subgroups, or heterogeneous trauma populations with variable imaging pathways and reference standards.

A key methodological issue is that CT is not routinely performed for all patients with foot and ankle trauma in real-world emergency care. Instead, CT is usually requested when there is persistent clinical suspicion despite normal radiographs, equivocal or suspicious radiographic findings, suspected complex or intra-articular injury, or a need for detailed fracture mapping. Consequently, studies based only on patients who undergo CT are vulnerable to selection bias, verification bias, and spectrum bias. In such cohorts, CT-confirmed fracture proportions and apparent diagnostic performance estimates should be interpreted as conditional findings within a selected CT population rather than as true diagnostic accuracy estimates for the general emergency trauma population [12,13,14,15].

The research perspective of the present study is therefore not to determine the population-level diagnostic accuracy of plain radiography. Rather, it is to describe real-world CT-confirmed fracture findings after plain radiography in a large selected cohort of adult isolated foot and ankle trauma patients who underwent CT as part of routine clinical care. This perspective is clinically relevant because emergency physicians and orthopedic surgeons often need to decide whether CT is likely to provide additional information after normal, suspicious, or definite radiographic findings. Large selected-cohort data stratified by radiographic category remain limited, particularly for isolated foot and ankle trauma.

The aim of this study was to evaluate CT-confirmed fracture proportions and the conditional diagnostic yield of CT according to plain radiography categories within a selected CT cohort of adult patients with isolated foot and/or ankle trauma. Secondary aims were to assess conditional apparent diagnostic performance of plain radiography within this selected cohort and to explore fracture-pattern-specific rates of additional CT contribution. We hypothesized that the CT-confirmed fracture proportion would be substantially higher among patients with suspicious radiographs than among those with normal radiographs, and that CT would identify additional fractures in selected patients whose plain radiographs did not demonstrate a definite fracture.

## 2. Materials and Methods

### 2.1. Study Design and Patient Selection

This retrospective single-center selected-cohort study was conducted among adult patients who presented to the Emergency Department of Van Training and Research Hospital, University of Health Sciences, Türkiye, with isolated foot and/or ankle trauma between 1 January 2023 and 31 December 2025. The study was conducted in accordance with the principles of the Declaration of Helsinki and was approved by the Non-Interventional Clinical Research Ethics Committee of Van Training and Research Hospital, University of Health Sciences, Türkiye (protocol code: GOKAEK/2025-10-07; approval date: 19 December 2025). Because of the retrospective design and use of anonymized data, the requirement for individual informed consent was waived. Written institutional permission was obtained for the use of hospital information system data.

The dataset was closed after completion of the study period and verification of all eligible encounters through 31 December 2025. No subsequent encounters were added after data closure. Consecutive patients who met the eligibility criteria were included. Inclusion criteria were as follows: age 18 years or older, isolated foot and/or ankle trauma, both plain radiography and CT performed during the same clinical visit, and availability of clinical and radiological data. Exclusion criteria were incomplete clinical data, incomplete imaging records, open fracture, pathological fracture, repeated visits, and multitrauma. Traffic accident-related trauma was excluded because such injuries are often associated with multiple-region trauma and different imaging algorithms.

Plain radiography and CT were requested as part of routine clinical care based on emergency physician assessment and orthopedic consultation when required. During the study period, CT was not mandated by a prospective study-specific algorithm. CT was generally obtained in cases with persistent focal clinical suspicion despite radiographs, radiographic uncertainty, suspected intra-articular or complex fracture, discordance between physical examination and radiographic assessment, or a need for preoperative fracture characterization. The study database did not contain systematic documentation of the individual components of the Ottawa ankle or foot rules; therefore, formal adherence to these decision rules could not be analyzed. No formal institutional CT-request protocol change was identified in the study records, but physician-level variability in CT ordering could not be quantified retrospectively.

### 2.2. Radiological Assessment

Radiological classifications were performed retrospectively, primarily on the basis of available radiology reports and accessible imaging records. The study was not designed as a formal blinded independent reader study with complete re-interpretation of all plain radiographs and CT examinations. Ambiguous or conflicting cases were re-evaluated by consensus between two orthopedic and traumatology specialists.

Plain radiographs were classified into three categories: normal, suspicious for fracture, and definite fracture. A radiograph was classified as normal when the report did not describe an acute fracture, suspicious fracture, cortical irregularity, or other finding considered suggestive of fracture. A radiograph was classified as suspicious when it did not establish a definite fracture diagnosis but included report wording or imaging findings such as “suspected fracture”, “possible fracture”, “cannot exclude fracture”, “suspicious cortical irregularity”, an equivocal radiolucent line, subtle cortical step-off, localized trabecular disruption insufficient for definite fracture diagnosis, questionable intra-articular extension, or localized soft-tissue swelling in the setting of persistent clinical concern. A radiograph was classified as definite fracture when the report explicitly described an acute fracture, cortical discontinuity, displaced fragment, intra-articular fracture extension, or a clearly visible fracture line.

A CT-confirmed fracture was defined as the presence of an acute fracture on CT. CT was treated as the comparative imaging reference within this selected CT cohort because it represented the most advanced imaging examination performed during the same clinical encounter. However, CT interpretation was performed in the routine clinical workflow and was not based on centralized blinded re-reading. The original CT readers may have had access to plain radiographs and clinical notes. Therefore, the CT reference standard was not fully independent, and the results should be interpreted as real-world selected-cohort CT yield rather than as a formal prospective diagnostic accuracy estimate.

Standard anatomic fracture-pattern categories were defined as calcaneus, lateral malleolus, medial malleolus, posterior malleolus, complex malleolar/syndesmotic injury, metatarsal, talus, navicular, midfoot/cuneiform/cuboid, distal tibia/fibula/pilon, and other. cases with CT-confirmed fractures in which the fracture-type field was blank or could not be reliably coded into a standard category were excluded from the fracture-pattern analysis and were reported separately.

### 2.3. Statistical Analysis

Continuous variables are presented as mean ± standard deviation or, when appropriate, median with interquartile range (IQR). Categorical variables are reported as numbers and percentages.

Plain radiography results were categorized as normal, suspicious for fracture, or definite fracture. A CT-confirmed fracture was defined as the presence of an acute fracture on CT.

The primary analysis focused on CT-confirmed fracture proportions according to plain radiography category. Indicators of additional CT contribution were calculated descriptively, including the proportion of patients not classified as definite fracture on plain radiography but found to have an acute fracture on CT. Confidence intervals for proportions were calculated using the exact binomial (Clopper–Pearson) method.

Conditional apparent diagnostic performance of plain radiography was evaluated only as a supplementary sensitivity analysis within the selected CT cohort. In the primary conditional model, only definite fractures on plain radiography were considered positive, whereas normal and suspicious radiographs were classified as negative. In the broader conditional model, suspicious and definite fracture radiographs were considered positive. These analyses were not intended to estimate the true sensitivity, specificity, positive predictive value, negative predictive value, or accuracy of plain radiography in the overall foot and ankle trauma population, because CT was not systematically performed in all trauma patients.

Fracture-pattern analysis was performed as an exploratory secondary analysis among cases with CT-confirmed fractures with available fracture-type classification. The additional diagnostic contribution of CT was defined as the number of cases with CT-confirmed fractures within each fracture pattern that had not been classified as definite fracture on plain radiography. Cases with CT-confirmed fractures with missing fracture-type classification were excluded from this analysis and reported separately.

No hypothesis testing or multivariable modeling was performed. Analyses were limited to descriptive statistics, proportion estimates, and selected-cohort conditional estimates. All analyses were conducted in Python 3.12 using pandas, scipy, and statsmodels packages.

### 2.4. Use of Generative Artificial Intelligence Tools

During preparation of this manuscript, ChatGPT-5 (OpenAI) was used for language editing, academic phrasing, formatting assistance, manuscript organization, and assistance with redesigning the figures from author-verified aggregate data. No generative artificial intelligence tool was used for study design, patient selection, data collection, statistical analysis, interpretation of radiographs or CT examinations, interpretation of the study results, or formulation of the scientific conclusions. All AI-assisted text and visual outputs were critically reviewed, verified, and edited by all authors, who take full responsibility for the accuracy, integrity, and scientific content of the manuscript.

## 3. Results

**Figure 1:**
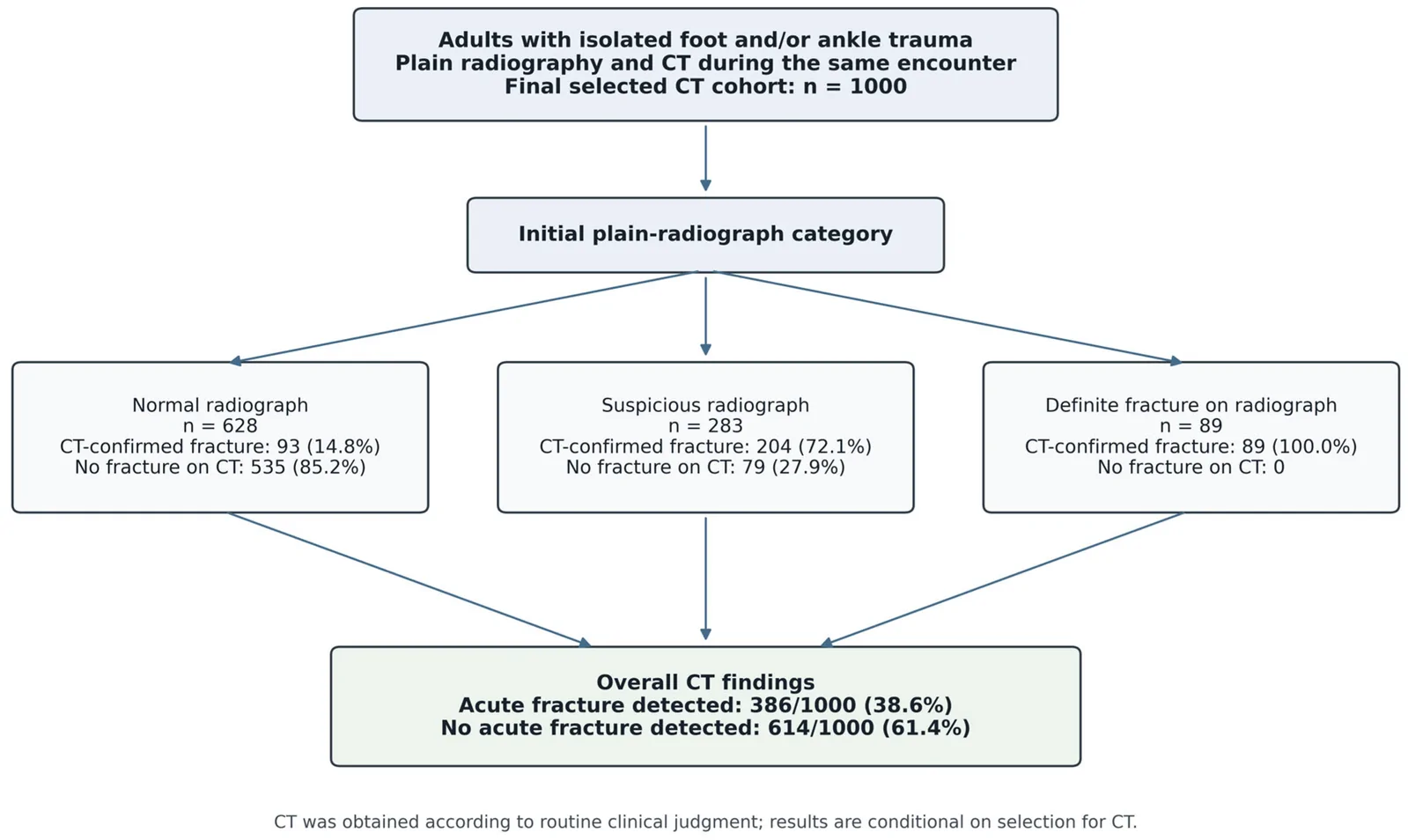
Flow diagram of the selected cohort, initial plain-radiograph categories, and CT findings. CT was obtained according to routine clinical judgment and was not systematically performed in all patients presenting with foot and ankle trauma.

The final analysis cohort consisted of 1000 adult patients who underwent both plain radiography and CT during the same clinical visit (Figure 1, Table 1). Acute fractures were detected on CT in 386 patients (38.6%), whereas no acute fracture was identified on CT in 614 patients (61.4%).

### 3.1. CT-Confirmed Fractures According to Plain-Radiograph Category

**Figure 2:**
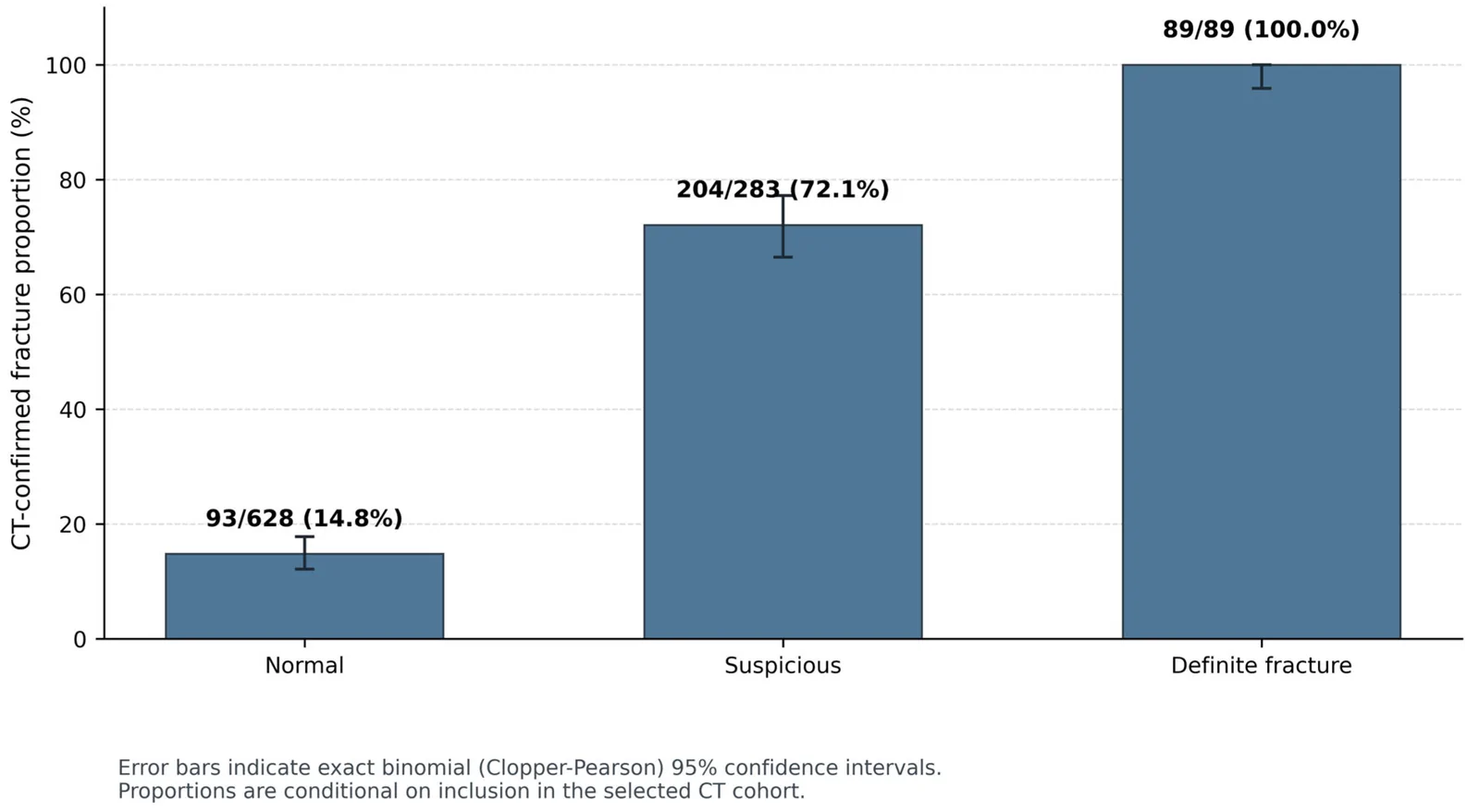
Proportion of CT-confirmed fractures according to the initial plain-radiograph category. Error bars indicate exact binomial (Clopper–Pearson) 95% confidence intervals. Proportions are conditional on inclusion in the selected CT cohort and should not be interpreted as population-level diagnostic accuracy estimates.

CT-confirmed fracture proportions according to the initial plain-radiograph category are shown in Table 2 and Figure 2. Among 628 patients with normal plain radiographs, CT confirmed an acute fracture in 93 (14.8%). Among 283 patients with suspicious radiographs, CT confirmed an acute fracture in 204 (72.1%). All 89 patients categorized as having a definite fracture on plain radiography had a fracture confirmed on CT. These findings are conditional on selection for CT and should not be interpreted as fracture prevalence in the overall emergency trauma population.

### 3.2. Additional Diagnostic Contribution and CT Examinations Without Detected Fracture

Indicators of additional diagnostic contribution and CT examinations without detected fracture are presented in Table 3. The proportion of patients not classified as definite fracture on plain radiography but found to have an acute fracture on CT was 29.7% (297/1000; 95% CI: 26.9–32.6). Patients with completely normal radiographs but fractures detected on CT accounted for 9.3% of the entire selected cohort (93/1000; 95% CI: 7.6–11.3). The proportion of CT examinations without detected fracture was 61.4% (614/1000; 95% CI: 58.3–64.4).

### 3.3. Conditional Apparent Diagnostic Performance Analysis

Conditional apparent diagnostic performance estimates are provided in Supplementary Table S1. In the strict conditional model, in which only definite fractures on plain radiography were considered positive, apparent sensitivity was 23.1% and apparent accuracy was 70.3% within the selected CT cohort. In the broader conditional model, in which suspicious radiographs were also considered positive, apparent sensitivity increased to 75.9% and apparent accuracy increased to 82.8%. These analyses provide clinical context regarding the suspicious radiograph category but should be interpreted strictly as conditional estimates within a highly selected CT cohort, not as true diagnostic accuracy parameters for plain radiography in the general foot and ankle trauma population.

### 3.4. Data Completeness for Fracture-Pattern Classification

Of 386 patients with CT-confirmed fractures, 301 (78.0%) could be classified into standard anatomic fracture-pattern categories. The fracture-type field was blank or missing in 85 cases (22.0%). Among cases with missing fracture-type data, 66 had normal radiographs, 18 had suspicious radiographs, and 1 had a definite fracture radiograph. These cases were excluded from the fracture-pattern analysis. Therefore, all fracture-pattern-specific findings should be interpreted as exploratory available-case analyses (Table 4).

### 3.5. Exploratory Fracture-Pattern Analysis

Exploratory fracture-pattern analysis was performed in 301 cases with CT-confirmed fractures with available fracture-type classification (Table 5). The additional CT contribution was most frequently observed in calcaneal fractures (67/68), metatarsal fractures (39/40), and posterior malleolar fractures (26/27). Lower proportions were observed for lateral malleolar fractures (25/65) and medial malleolar fractures (10/27). Very small subgroups, including navicular fractures and distal tibia/fibula/pilon fractures, are reported primarily as raw counts because percentage estimates are unstable when sample sizes are limited. These findings are descriptive and hypothesis-generating.

## 4. Discussion

In this retrospective selected CT cohort of adult patients with isolated foot and/or ankle trauma, CT-confirmed fracture proportions varied substantially according to the initial plain radiography category. CT identified acute fractures in 14.8% of patients whose radiographs were categorized as normal and in 72.1% of patients whose radiographs were categorized as suspicious. These findings support the clinical relevance of persistent clinical suspicion and equivocal radiographic findings when selecting patients for CT. However, because CT was performed only in selected patients, the results should not be interpreted as the prevalence of occult fracture or as the diagnostic accuracy of plain radiography in the overall emergency trauma population.

The present study should be interpreted from a selected-cohort imaging-yield perspective. Plain radiography remains the first-line imaging modality in foot and ankle trauma [1,2,16]. CT is not indicated for all patients, but it may provide additional information when radiographs are normal despite persistent focal symptoms, when radiographs are equivocal, or when complex/intra-articular fracture morphology needs clarification [1,5]. The observed 14.8% CT-confirmed fracture proportion among patients with normal radiographs likely reflects enrichment by clinical selection rather than a generalizable occult fracture rate.

The findings align with prior literature indicating that CT can contribute to the detection or characterization of fractures in complex foot and ankle regions. Previous studies have reported clinically relevant differences between radiographs and CT for Lisfranc injuries, talar injuries, calcaneal fractures, posterior malleolar fractures, and malleolar fracture planning [6,7,8,9,10,11].

The present study adds to this literature by stratifying CT-confirmed fracture findings according to initial radiographic category in a relatively large real-world cohort of adult isolated foot and ankle trauma patients who underwent CT. This approach reflects everyday emergency and orthopedic decision-making more closely than a purely anatomic subgroup study, but it cannot replace a prospective diagnostic accuracy design.

The suspicious radiograph category appears clinically meaningful. When suspicious radiographs were treated as positive in the conditional supplementary analysis, apparent sensitivity increased substantially compared with the strict model. This finding suggests that equivocal radiographic language may identify a subgroup with a high probability of a CT-confirmed fracture in selected patients. However, the category remains partly subjective because it was derived retrospectively from original radiology reports and available imaging records rather than from standardized prospective criteria. For this reason, suspicious radiographs should be interpreted as a practical clinical category rather than a reproducible diagnostic threshold.

Fracture-pattern findings should be interpreted with particular caution. Although additional CT contribution was frequently observed in calcaneal, metatarsal, and posterior malleolar fractures, this analysis was limited by missing fracture-type classification in 22.0% of cases with CT-confirmed fractures and by small sample sizes in several anatomic subgroups. Therefore, these results are best viewed as descriptive and hypothesis-generating. They should not be used to infer stable fracture-specific performance estimates or to recommend CT routinely for specific fracture patterns without considering clinical context.

From a clinical perspective, the study supports a selective approach to CT rather than indiscriminate advanced imaging. CT may be useful when clinical examination, swelling, weight-bearing inability, or focal tenderness remains discordant with normal radiographs; when radiographs are suspicious but not definitive; or when fracture morphology may influence treatment planning. At the same time, the high proportion of CT examinations without detected fracture underscores the need for careful patient selection and prospective evaluation of imaging algorithms.

Future research should use prospective designs with predefined CT indications, systematic documentation of decision rules such as the Ottawa ankle and foot rules, independent blinded image review, and interobserver reliability assessment. Such studies should include either systematic verification in broader trauma populations or appropriate methods to address partial verification bias. Prospective multicenter cohorts would also help clarify whether selected-cohort CT-confirmed fracture proportions are consistent across institutions, clinicians, scanners, and emergency care pathways.

This study has several limitations. First, it was retrospective and single-center. Second, because only patients who underwent CT were included, selection bias, verification bias, and spectrum bias are likely [14,15]. The 14.8% CT-confirmed fracture proportion among patients with normal radiographs is therefore mathematically and clinically enriched relative to the general emergency trauma population. Third, CT request indications were not standardized by a prospective algorithm, and physician-level variability in CT ordering could not be quantified. Fourth, radiograph classification was based primarily on original radiology reports and available imaging records, and the distinction between normal and suspicious radiographs may have been influenced by report wording and clinical documentation. Fifth, imaging studies were not fully re-evaluated by independent blinded readers, no formal interobserver reliability analysis was performed, and CT interpretations were not a completely independent reference standard because original CT readers may have had access to radiographs and clinical notes. Sixth, fracture-pattern classification was missing in 85 of 386 cases with CT-confirmed fractures (22.0%), and several subgroup analyses had limited sample sizes. These limitations reinforce that the findings represent real-world selected-cohort CT yield rather than true diagnostic accuracy estimates.

## 5. Conclusions

Within a selected CT cohort of adult isolated foot and ankle trauma patients, CT identified additional acute fractures in a subset of cases that could not be definitively classified as fractures on plain radiography. The CT-confirmed fracture proportion was particularly high among patients with suspicious radiographs, supporting the clinical relevance of equivocal radiographic findings when CT is being considered. However, because CT was not systematically performed in all trauma patients and because imaging assessment was retrospective and not independently blinded, these findings should be interpreted as selected-cohort CT yield rather than true diagnostic accuracy or general-population occult fracture prevalence. Prospective studies with standardized imaging algorithms, independent image review, and systematic documentation of clinical decision rules are needed to define advanced imaging strategies more precisely in foot and ankle trauma.

## Figures and Tables

**Table 1:** Baseline clinical and imaging characteristics of the cohort.

Variable	Value
Total patients	1000
Age, years, mean ± SD	39.7 ± 12.2
Age, years, median (IQR)	38 (32–48)
Sex: female	348 (34.8%)
Sex: male	652 (65.2%)
Trauma mechanism: sprain	343 (34.3%)
Trauma mechanism: fall	460 (46.0%)
Trauma mechanism: sports injury	25 (2.5%)
Trauma mechanism: high energy	172 (17.2%)
Inability to bear weight	728 (72.8%)
Ecchymosis	317 (31.7%)
Swelling: absent	269 (26.9%)
Swelling: mild	707 (70.7%)
Swelling: marked	24 (2.4%)
Plain radiography result: normal	628 (62.8%)
Plain radiography result: suspicious	283 (28.3%)
Plain radiography result: definite fracture	89 (8.9%)
CT result: no fracture detected	614 (61.4%)
CT result: acute fracture detected	386 (38.6%)

Data are presented as n (%) unless otherwise indicated. IQR, interquartile range; CT, computed tomography.

**Table 2:** Cross-tabulation of plain radiography and CT results.

Plain Radiography Result	No Fracture Detected on CT	CT-Confirmed Fracture	Total	CT-Confirmed Fracture Proportion
Normal	535	93	628	14.8%
Suspicious	79	204	283	72.1%
Definite fracture	0	89	89	100.0%
Total	614	386	1000	38.6%

This table shows the relationship between plain radiography and CT within the selected CT cohort.

**Table 3:** Indicators of additional diagnostic contribution and CT examinations without detected fracture.

Indicator	Numerator/Denominator	Rate	95% CI
Overall fracture detection rate on CT	386/1000	38.6%	35.6–41.7
No definite fracture on radiography but fracture detected on CT	297/1000	29.7%	26.9–32.6
Normal radiograph and fracture detected on CT	93/1000	9.3%	7.6–11.3
CT-confirmed fracture proportion among patients with normal radiographs	93/628	14.8%	12.1–17.8
CT-confirmed fracture proportion among patients with suspicious radiographs	204/283	72.1%	66.5–77.2
CT-confirmed fracture proportion among definite fracture radiographs	89/89	100.0%	95.9–100.0
CT examinations without detected fracture	614/1000	61.4%	58.3–64.4
Normal radiograph and no fracture detected on CT	535/1000	53.5%	50.4–56.6
Suspicious radiograph and no fracture detected on CT	79/1000	7.9%	6.3–9.7

CI, confidence interval. Exact binomial (Clopper–Pearson) 95% CIs were calculated for proportions.

**Table 4:** Fracture-type classification status among cases with CT-confirmed fractures.

Variable	n	Proportion Among CT-Confirmed Fracture Cases
Total CT-confirmed fracture patients	386	100.0%
Classifiable into standard fracture-pattern categories	301	78.0%
Fracture-type field blank/missing	85	22.0%
Missing fracture type: normal radiograph	66	17.1%
Missing fracture type: suspicious radiograph	18	4.7%
Missing fracture type: definite fracture radiograph	1	0.3%
Total check	386	100.0%

The total check row sums to the total number of cases with CT-confirmed fractures. Cases with blank fracture-type fields were not included in the fracture-pattern analysis.

**Table 5:** Plain radiography categories and additional CT contribution in the exploratory fracture-pattern analysis.

Fracture Pattern	Total	Normal Radiograph	Suspicious Radiograph	Definite Fracture Radiograph	Additional CT Contribution, n/N (%)
Calcaneus	68	15	52	1	67/68 (98.5%)
Lateral malleolus	65	4	21	40	25/65 (38.5%)
Complex malleolar/syndesmotic	50	0	24	26	24/50 (48.0%)
Metatarsal	40	2	37	1	39/40 (97.5%)
Posterior malleolus	27	1	25	1	26/27 (96.3%)
Medial malleolus	27	1	9	17	10/27 (37.0%)
Midfoot/cuneiform/cuboid	6	1	5	0	6/6; small subgroup
Talus	10	1	9	0	10/10; small subgroup
Navicular	4	1	3	0	4/4; small subgroup
Distal tibia/fibula/pilon	4	1	1	2	2/4; small subgroup

Fracture-pattern analysis was performed only in cases with CT-confirmed fractures with available fracture-type classification. Additional CT contribution = normal radiograph + suspicious radiograph within the corresponding fracture pattern. Small subgroups should be interpreted cautiously, and raw frequencies should be prioritized over percentages.

## Data Availability

The data presented in this study are available from the corresponding author upon reasonable request. Data sharing will be subject to institutional approval and applicable privacy regulations.

## Supplementary Materials

The following supporting information can be downloaded at https://www.mdpi.com/article/10.3390/jcm15166214/s1, Supplementary Table S1: Conditional apparent diagnostic performance of plain radiography within the selected CT cohort.

## Abbreviations

CT, computed tomography; CI, confidence interval; IQR, interquartile range; ACR, American College of Radiology.
