# Supplementary material for: Additional Fracture Detection by Computed Tomography After Plain Radiography in Adults with Isolated Foot and Ankle Trauma: A Retrospective Selected-Cohort Study
Source: J Clin Med. 2026 Aug 11;15(16):6214. doi: 10.3390/jcm15166214 (PMC13513773; doi:10.3390/jcm15166214)
Supplement: Supplementary file 1 [file jcm-15-06214-s001.zip › jcm-4379261-supplementary.pdf]

Supplementary Table S1. Conditional apparent diagnostic performance of plain radiography within the selected CT cohort

Metric	Strict conditional model	95% CI	Broad conditional model	95% CI
Apparent sensitivity	23.1%	18.9-27.6	75.9%	71.3-80.1
Specificity	100.0%	99.4-100.0	87.1%	84.2-89.7
Positive predictive value	100.0%	95.9-100.0	78.8%	74.3-82.8
Negative predictive value	67.4%	64.2-70.4	85.2%	82.2-87.9
Accuracy	70.3%	67.4-73.1	82.8%	80.3-85.1

Strict conditional model: only radiographs classified as definite fracture were considered positive. Broad conditional model: radiographs classified as suspicious or definite fracture were considered positive.

These values are conditional estimates among patients who underwent CT. Because CT was not systematically performed in the overall foot and ankle trauma population, these estimates should not be interpreted as population-level diagnostic accuracy parameters.
